# Epilepsy With Auditory Features: From Etiology to Treatment

**DOI:** 10.3389/fneur.2021.807939

**Published:** 2022-01-27

**Authors:** Alessandro Furia, Laura Licchetta, Lorenzo Muccioli, Lorenzo Ferri, Barbara Mostacci, Stefania Mazzoni, Veronica Menghi, Raffaella Minardi, Paolo Tinuper, Francesca Bisulli

**Affiliations:** ^1^Department of Biomedical and NeuroMotor Sciences, Alma Mater Studiorum-University of Bologna, Bologna, Italy; ^2^Istituto di Ricerca e Cura a Carattere Scientifico (IRCCS) Istituto delle Scienze Neurologiche di Bologna, Reference Center for Rare and Complex Epilepsies, Bologna, Italy

**Keywords:** auditory hallucinations, epilepsy, *DEPDC5*, mTOR, *LGI1*, aphasic seizures

## Abstract

Epilepsy with auditory features (EAF) is a focal epilepsy belonging to the focal epileptic syndromes with onset at variable age according to the new ILAE Classification. It is characterized by seizures with auditory aura or receptive aphasia suggesting a lateral temporal lobe involvement of the epileptic discharge. Etiological factors underlying EAF are largely unknown. In the familial cases with an autosomal dominant pattern of inheritance several genes have been involved, among which the first discovered, *LGI1*, was thought to be predominant. However, increasing evidence now points to a multifactorial etiology, as familial and sporadic EAF share a virtually identical electro-clinical characterization and only a few have a documented genetic etiology. Patients with EAF usually have an unremarkable neurological examination and a good response to antiseizure medications. However, it must be underscored that total remission might be lower than expected and that treatment withdrawal might lead to relapses. Thus, a proper understanding of this condition is in order for better patient treatment and counseling. Further studies are still required to further characterize the many facets of EAF.

## Introduction

Epilepsy with auditory features (EAF) is a focal epilepsy included in the group of focal syndromes with onset at variable age in the new ILAE Classification, besides focal epilepsy with variable foci (FFEAF) and sleep-related hypermotor epilepsy (SHE), with which it shares some genetic aspects (1). The new simplified nomenclature aims to overcome the previous, possibly misleading, use of two different names for the same syndrome, namely autosomal dominant lateral temporal lobe epilepsy (ADLTLE) and autosomal dominant partial epilepsy with auditory features (ADPEAF), and to acknowledge this entity also in a non-familial context/pattern.

EAF phenotype is characterized by seizures with auditory symptoms or aphasia, suggesting a lateral temporal lobe involvement.

Auditory symptoms have long been associated with epilepsy, with the first description of such presentation dating as early as 1,883 (2). More generally, auditory hallucinations may be classified as simple (hearing a monotone sound such as humming or buzzing as in tinnitus) and complex (hearing voices or music), in which case they can be defined as auditory verbal hallucinations (3). On this basis, it has also been proposed that several historical figures were affected by epilepsy with auditory hallucinations (4, 5), including Fyodor Dostoevsky, whose known epilepsy might have first manifested in childhood with verbal hallucinations, as illustrated in his autobiographical work “The Peasant Marey” (5).

Aside from reasons of historical interest, recognizing the epileptic origin of auditory phenomena/symptoms is key for both the neurologist and other clinicians. Quite commonly, auditory features in epilepsy are misdiagnosed as psychiatric disease, most often schizophrenia, as shown in several reports (6–8).

Even within the field of epileptology, appreciating the clinical features of EAF, an entity whose prevalence might be higher than expected, is paramount to a proper diagnosis and adequate treatment.

Additionally, recognizing the propensity of lateral temporal seizures to evolve into focal to bilateral tonic-clonic ones helps avoid the pitfall of misdiagnosing focal epilepsy as an idiopathic generalized entity, with consequent therapeutic implications.

## History and Nomenclature From 1995 to 2021

In 1995, on the same *Nature Genetics* volume in which a genetic basis for SHE was first described (9) Ottman et al. published a study in which they identified a locus on chromosome 10q associated with focal epilepsy in 11 members of a large family (10). This entity showed autosomal dominant transmission with high penetrance. Of the 11 members affected, 55% reported auditory symptoms: all had focal to bilateral tonic-clonic seizures. Despite being identified only relatively recently, EAF stands as an example of how a paradigmatic shift allowed to focus on the genetic basis of focal, and not only generalized, epilepsy.

This first observation was then corroborated in 1999 by Poza et al., who described 19 individuals from a Basque family suffering from a similar form of focal epilepsy, even in this case linked to chromosome 10q (11). However, compared to the earlier study, the members also showed other sensory symptoms, such as visual hallucinations. A new term was proposed for such epilepsy, autosomal dominant lateral temporal lobe epilepsy (ADLTE), to highlight the supposed anatomical origin instead of mesial temporal epilepsy.

Subsequently, Winawer et al. further elaborated their clinical description of the 1999 family (12). In particular, the Authors introduced an alternative name, i.e., autosomal dominant partial epilepsy with auditory features (ADPEAF), to emphasize the high prevalence of ictal auditory symptoms.

A decisive turn in the characterization of ADLTE/ADPEAF was made in 2002, when Kalachicov et al. identified the first causative gene of ADLTE, *LGI1*, on chromosome 10q24. In particular, they identified five different pathogenic *LGI1* variants, of which three were frameshift, one was missense and the last one was a splice site variant (13). *LGI1* mutations were also associated to temporal lobe epilepsy with aphasic seizures (14), demonstrating that other ictal manifestations are to be considered in the phenotypic spectrum of temporal lobe epilepsy with auditory features. The putative role of *LGI1* in both normal and disease states will be discussed in the sections below.

In 2015, causative variants of a second gene, *RELN*, were found in seven families (15), followed by identifications of other genes, including *DEPDC5, MICAL-1, CNTNAP2*, and *SCN1*A (16–18).

In 2021, the Nosology and Definition Task Force of the ILAE, by introducing position papers for definition and new nomenclature of epileptic syndromes (1), proposed the term Epilepsy with Auditory Features (EAF) to encompass both ADLTE and ADPEAF, also defining the inherited form as Familial EAF (FEAF).

## Clinical Features and Investigative Findings

Since the 1989 ILAE classification of epilepsies and epileptic syndromes (19), temporal lobe epilepsy has been divided into mesial and lateral forms: the most salient features distinguishing the two are shown in Table 1. It can be noticed that auditory symptoms are a prominent feature of lateral temporal epilepsy, therefore being of great clinical help.

**Table 1:** Differences between mesial and lateral temporal lobe epilepsy.

	**Mesial temporal lobe epilepsy**	**Lateral temporal lobe epilepsy**
Localization	Hippocampus, parahippocampal gyrus, amygdala, entorhinal cortex	Temporal neocortex
Clinical presentation [adapted from (20)]	•Autonomic (epigastric/abdominal discomfort), cognitive (déjà vu/jamais vu), emotional (fear) seizures are common •Olfactory and gustatory seizures are possible •Behavioral arrest with oral/manual automatisms •Contralateral upper limb dystonia with contralateral head and eye version •Longer seizure duration	•Sensory hallucinations (e.g., auditory) are prominent •Faster onset of impaired awareness •Shorter seizure duration
Seizure evolution in focal to bilateral tonic-clonic	Less frequent	More frequent
Interictal EEG abnormalities	More frequent	Less frequent
Drug resistance	More frequent	Less frequent

The clinical features of EAF are summarized in Table 2.

**Table 2:** Clinical features of EAF [adapted from (21)].

Family history	Might be positive for febrile seizures, rarely for intellectual disability or psychiatric disorders
Personal history	Might be positive for febrile seizures
Interictal EEG	Usually normal, in some cases focal (temporal) or diffuse epileptiform abnormalities
Seizure semiology	Aura: might be auditory or aphasic (receptive/global aphasia) Auditory hallucinations: simple/complex Focal to generalized tonic-clonic seizures are possible Reflex seizures from hearing a sound could be present

Considering that isolated auditory symptoms, especially when these are simple, might not be adequately recognized as pertaining to a disease and in particular epilepsy, a precise estimate of the incidence of EAF is currently not available.

Several studies have investigated the clinical presentation of both sporadic and familial EAF (22–24): notably, it appears that there are no significant clinical differences between the two forms (22).

It is now understood that EAF is an epileptic syndrome whose onset is not age-related although it is more frequent in the second and third decade of life.

Patients do not show abnormalities at the physical or neurological exam and in their personal history of birth and development.

As the name suggests, the key feature of EAF consists of focal aware sensory seizures with auditory symptoms, which can be both simple or complex. Patients can also present with focal aware cognitive seizures, i.e., receptive aphasia. Oher sensory seizures (most often visual) may also occur. Focal to bilateral tonic-clonic or focal impaired awareness seizures are possible. Most importantly, these might be the first seizures to be noticed and reported. These seizures frequently occur during sleep and the focal signs, namely auditory features, may be easily missed, possibly leading to an idiopathic generalized epilepsy misdiagnosis (1).

The most important entity in differential diagnosis with FEAF is FFEVF, which can also present with auditory seizures. However, diagnostic criteria for EAF require that all affected family members present with auditory symptoms, as opposed to isolated family members in FFEVF.

Simple and complex auditory hallucinations pertaining to other disorders (most commonly, tinnitus or schizophrenia) can be excluded by careful clinical observation and history gathering: the sound of tinnitus is far more durable than the seizure of EAF, while schizophrenic hallucinations are quite complex and often accompanied by other features of this psychiatric disease.

**Figure 1:**
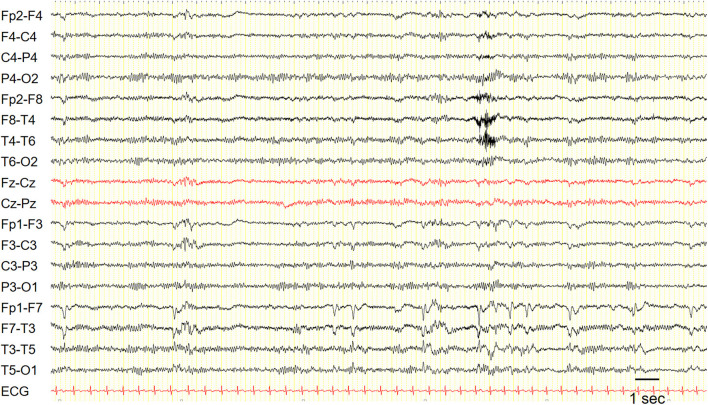
EEG in a patient with epilepsy with auditory features showed recurrent epileptiform discharges in the left fronto-temporal region. Sensitivity: 7 uV/mm.

Interictal EEG is often unremarkable, but it may show focal temporal sharp waves or spikes (Figure 1).

**Figure 2:**
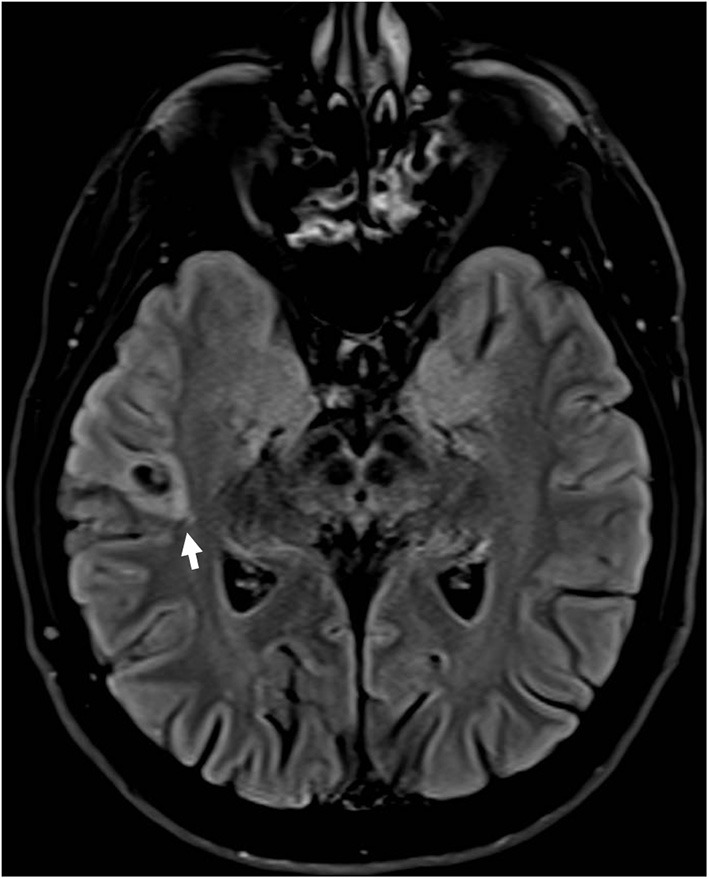
Brain MRI (FLAIR; axial view) in a patient with drug-resistant epilepsy with auditory features showed a small hyperintense lesion in the right lateral temporal cortex surrounded by an area of signal suppression and hyperintensity of the adjacent brain tissue, consistent with a glioneuronal tumor.

Some patients with EAF may have an underlying lesion on brain MRI (Figure 2); however, in contrast to what described in early studies (23), also reporting cases associated with *LGI1* mutations (24), this occurrence is infrequent (25). This notwithstanding, high-resolution MRI remains a key investigational procedure in patients with EAF in order to exclude a lesional etiology, a finding which may have remarkable therapeutic implications, notably epilepsy surgery in selected patients. In some cases, the absence of an underlying lesion might represent a false negative finding related to insufficient MRI resolution; however, a non-lesional cause should become the prime suspect. Some cases with lesional EAF may have an underlying mutation, such as GATOR1-related focal cortical dysplasia; however, as discussed below, the genetics of EAF has not been fully elucidated yet, and genotype-phenotype correlations are difficult to establish.

## Genetics: from *LGI1* to mTOR genes

The genes involved with EAF are presented in Table 3. These will now be reviewed in more detail.

**Table 3:** Genes linked to EAF.

*LGI1*	Identified in 1995 Modulation of AMPA-related neurotransmission
*RELN*	Identified in 2017 Neuronal migration and plasticity
*DEPDC5*	Identified in 2015 Role in mTOR pathway and cortical dysplasia
*CNTNAP2*	Identified in 2018 Encodes a neurexin protein
*SCN1A*	Identified in 2019 Related to generalized epilepsy with febrile seizures plus (GEFS+): associated to EAF in (26)
*MICAL-1*	Identified in 2018 Cytoskeletal regulation Not currently confirmed

## LGI1

Leucine-rich, glioma inactivated protein 1 (also known as epitempin) is a protein encoded by the *LGI1* gene on chromosome 10q24. Initially discovered by Chernova and colleagues in 1998 (27), *LGI1* is a predominantly neuronal protein, whose downregulation was first implicated in the pathogenesis of malignant brain neoplasms (in particular glioblastoma multiforme) as its own name suggests. Thus, its putative role was believed to be tumor suppression. However, *LGI1* has been implicated in various other processes, including neuronal transmission (28) and development (29): therefore, its role remains to be fully elucidated.

Epitempin binds to its receptor, ADAM22 (part of the transmembrane ADAM metalloprotease family), forming a complex which regulates neurotransmission through inhibition of the AMPA receptor (30, 31).

More than 40 pathogenic variants of *LGI1* have been described in EAF (30). These mutations, which all lead to loss of function, point to a likely mechanism of *LGI1* haploinsufficiency in causing EAF, which would also explain the autosomal dominant pattern of inheritance. Moreover, the temporal origin of seizures and the present evidence regarding epitempin suggest that abnormal neuronal migration with the formation of dysfunctional circuits might be the underlying mechanism linking LGI1 to epilepsy (30).

Also, antibodies against epitempin disrupting the LGI1-ADAM22 complex cause a rare form of autoimmune encephalitis, characterized in some cases by peculiar faciobrachial dystonic seizures (FBDS) preceding the full-fledged clinical picture of cognitive dysfunction, memory impairment, and, eventually, tonic-clonic seizures. Interestingly, FBDS respond exquisitely well to immunotherapy (32). Future research might uncover a possible relation, currently not clear, between these close but different entities.

## *DEPDC5* and the mTOR Pathway

The *DEPDC5* (DEP domain containing 5, *GATOR1* subcomplex subunit) gene has been associated to all the three epileptic syndromes with onset at variable age, thus comprising SHE, FFEVF and EAF, as well as mesial temporal lobe epilepsy (16, 33). The *GATOR1*-complex is a negative controller of the mTOR (mammalian target of rapamycin) pathway, a molecular pathway fundamental in several functions key to cell survival, such as proliferation and growth (34). Developmental brain abnormalities caused by pathogenic variants in *DEPDC5* and genes coding for other component of the mTOR pathway might justify its association with focal cortical dysplasia (FCD), which according to its anatomical position might cause a wide array of different *DEPDC5-*related focal epilepsies. However, not all individuals carrying *DEPDC5* variants show FCD on brain MRI, suggesting that, in case of missense variants, brain structural lesions are caused by and additional brain somatic mutation, according to a double hit mechanism. *DEPDC5*-related EAF was first reported by Pippucci et al. in a family with few affected indivuals showed EAF as predominant epilepsy phenotype (16). It can be argued if the family studied represents instead a FFEVF pedigree, due to its small size. This notwithstanding, if the relation to EAF is established, mTOR targeting with specific drugs might be useful in the subset of patients with *DEPDC5*-related EAF.

## Other Genes *(RELN, MICAL-1, CNTNAP2, SCN1A)*

The *RELN* gene on chromosome region 7q22 encodes reelin, a large secreted protein that modulates both neuronal migration in the embryonal stage and neuronal plasticity in adult life (26, 35). Reelin has also been implicated in several neurological disorders, comprising lissencephaly and Alzheimer's disease (36, 37). RELN mutations were recently discovered in families affected by EAF, but no differences in clinical phenotype with LGI1-related epilepsy could be found (22).

The *MICAL-1* (Microtubule Associated Monooxygenase, Calponin and LIM Domain Containing 1) gene, on chromosome 6q21, encodes for a protein regulating the actin cytoskeleton. Likely pathogenic variants were identified in two unrelated EAF family in one study (18), but the association of the *MICAL1* gene with EAF is still under scrutiny. Other described mutations include *CNTNAP2* (Contactin-associated protein-like 2) and *SCN1A* (sodium channel, voltage-gated, type I, alpha subunit), which might point that EAF pertains to the spectrum of *SCN1A*-related epilepsy (16, 38, 39).

In conclusion, while it is commonly believed that *LGI1* and *RELN* together make up for most of the cases of FEAF, a recent study has suggested that truly genetically determined cases are rare (21) and that etiology could be multifactorial. Therefore, along with further discoveries of new genetic mutations underlying EAF, a more precise characterization of known defects is needed, too.

## Therapy

EAF is considered a syndrome with a good response to anti-seizure medications used for focal epilepsy, such as carbamazepine in monotherapy (40). However, patients might refuse pharmacological therapy for a disease perceived as trivial, especially in milder presentations. Surgery might be employed instead in resistant cases. When misdiagnosed as idiopathic generalized epilepsy, EAF might be treated with drugs that are not optimal (i.e., phenobarbital or valproate), leading to poor response and to the risk of mislabeling a treatable epileptic syndrome as drug-resistant.

Another aspect to consider is that drug withdrawal often leads to clinical relapses, not always responding to reinitiation of treatment (40). These aspects should be stressed when discussing the therapeutic options with the patient.

## Prognosis

Studies concerning the prognosis of both sporadic and familial EAF are only a few and mostly consist of reports of isolated families with few individuals.

The largest cohort of EAF patients was collected in a study by our group (40), in which 123 EAF patients (mostly sporadic) were followed with a median time of 11 years, using as primary endpoint total remission (seizure-free period of more than 5 years).

The three key factors negatively affecting remission in the cohort were the age onset (<10 years), complex auditory hallucinations, and focal EEG epileptiform abnormalities. A paramount aspect to be considered is the significant heterogeneity of the cohort in terms of phenotype's severity, from mild to refractory cases only referable by surgery, which might also explain why the total remission rate in this study was lower than expected (34.1%).

## Conclusions

The research field on EAF still presents several questions and challenges to be addressed. First, the actual prevalence of this syndrome must be thoroughly investigated.

Second, more work should be done on genetics, perhaps shifting the original paradigm from a disease dominated by LGI1 mutations to a multifactorial syndrome that can be stratified in different groups.

Following this direction, it could be possible in the future to separate patients into favorable groups where pharmacological treatment is useful and groups where epilepsy is strongly resistant, with surgery as the only viable option. In this sense, as evidence is still lacking, more effort should be spent on characterizing *DEPDC5*-related EAF and understanding the extent of mutation-associated cortical dysplasia.

In conclusion, EAF demonstrates how much progress has been made in the field of genetics in focal epilepsy, historically considered the paradigm of lesional/suspected lesional epilepsies, and how much more can be achieved in the future.

## Author Contributions

AF wrote the first draft of the manuscript. RM and LL reviewed the literature on the genetic aspects of EAF and provided corrections. LM, LF, and BM reviewed the manuscript concerning the section on clinical presentation, treatment, and prognosis. SM, VM, RM, PT, and FB reviewed and corrected the final version of the manuscript and also providing useful insight for improving it. All authors contributed to the article and approved the submitted version.

## Funding

This study was funded by the Istituto di Ricerca e Cura a Carattere Scientifico (IRCCS) Istituto delle Scienze Neurologiche di Bologna.

## Conflict of Interest

The authors declare that the research was conducted in the absence of any commercial or financial relationships that could be construed as a potential conflict of interest.

## Publisher's Note

All claims expressed in this article are solely those of the authors and do not necessarily represent those of their affiliated organizations, or those of the publisher, the editors and the reviewers. Any product that may be evaluated in this article, or claim that may be made by its manufacturer, is not guaranteed or endorsed by the publisher.
